# Red blood cell distribution width is associated with increased interactions of blood cells with vascular wall

**DOI:** 10.1038/s41598-022-17847-z

**Published:** 2022-08-11

**Authors:** Sharan Ananthaseshan, Krzysztof Bojakowski, Mariusz Sacharczuk, Piotr Poznanski, Dominik S. Skiba, Lisa Prahl Wittberg, Jordan McKenzie, Anna Szkulmowska, Niclas Berg, Piotr Andziak, Hanna Menkens, Maciej Wojtkowski, Dorota Religa, Fredrik Lundell, Tomasz Guzik, Zbigniew Gaciong, Piotr Religa

**Affiliations:** 1 Department of Medicine, Solna, Karolinska Institute, Stockholm, Sweden; 2 Department of Internal Medicine, Hypertension and Vascular Diseases, Medical University of Warsaw, 1a Banacha Street, 02-097 Warsaw, Poland; 3 2nd Vascular Surgery and Angiology Department, Centre of Postgraduate Medical Education, Warsaw, Poland; 4 Department of Experimental Genomics, Institute of Genetics and Animal Biotechnology, Polish Academy of Sciences, Jastrzebiec, Poland; 5 KTH Mechanics, Royal Institute of Technology, Stockholm, Sweden; 6 AM2M Ltd. L.P., Torun, Poland; 7 Institute of Physics, Nicolaus Copernicus University, Torun, Poland; 8 NVS, Karolinska Institute, Stockholm, Sweden; 9 Institute of Cardiovascular and Medical Sciences, University of Glasgow, Glasgow, UK

**Keywords:** Cardiovascular biology, Blood flow

## Abstract

The mechanism underlying the association between elevated red cell distribution width (RDW) and poor prognosis in variety of diseases is unknown although many researchers consider RDW a marker of inflammation. We hypothesized that RDW directly affects intravascular hemodynamics, interactions between circulating cells and vessel wall, inducing local changes predisposing to atherothrombosis. We applied different human and animal models to verify our hypothesis. Carotid plaques harvested from patients with high RDW had increased expression of genes and proteins associated with accelerated atherosclerosis as compared to subjects with low RDW. In microfluidic channels samples of blood from high RDW subjects showed flow pattern facilitating direct interaction with vessel wall. Flow pattern was also dependent on RDW value in mouse carotid arteries analyzed with Magnetic Resonance Imaging. In different mouse models of elevated RDW accelerated development of atherosclerotic lesions in aortas was observed. Therefore, comprehensive biological, fluid physics and optics studies showed that variation of red blood cells size measured by RDW results in increased interactions between vascular wall and circulating morphotic elements which contribute to vascular pathology.

## Introduction

Modern automated hematology instruments allow for simultaneous measurement of number and size of red blood cells (RBC). Based on red cell volume distribution curve the degree of variation in RBC size (i.e. anisocytosis) can be quantitated and expressed as red blood cell distribution width (RDW) which was traditionally used to diagnose different types of anemia. However, during last decade, numerous studies showed that high RDW strongly correlates with cardiovascular and all-cause morbidity and mortality in healthy populations as well as patients with various clinical conditions. This includes cardiovascular diseases (hypertension, carotid atherosclerosis, coronary heart disease, heart failure, atrial fibrillation, pulmonary embolism, ischemic stroke), cancer, diabetes, acute pancreatitis, liver and kidney failure, sepsis, Parkinsonism and COVID-19^1,2^. High RDW value has been reported to be a strong predictor of unfavorable clinical outcomes independent of concomitant conditions and could be used for risk stratification in certain groups of patients.

The specific mechanism for association of high RDW and health outcomes has not been identified. RDW may be a nonspecific marker of general illness and degree of inflammatory process. Excessive oxidative stress accelerates senescence of erythrocytes and erythrophagocytic clearance^3^. Moreover, during inflammatory process, the production of neutrophils and platelets increases that may slow development of erythroid lineage. Since the volume of RBC decreases over its lifespan, changes in both production kinetics and/or clearance of erythrocytes may modify RDW value^4^.

Recently, proteomics data analysis along with RDW measurement identified potential protein pathways connecting high RDW with all-cause mortality in prospectively observed aging population^5^. Previously, we have also identified mechanism linking certain proteins like VEGF with altered RBC production in experimental cancer model^6^. We have also found that infection with cytomegalovirus (CMV) inhibits erythropoietin production by blocking renal expression of hypoxia-inducible factor 2α (HIF2α)^7^.

The majority of clinical complications associated with high RDW results from vascular pathology, mostly atherothrombotic incidents due to platelet activation. Under normal physiological conditions, in flowing blood, RBCs tend to concentrate in the axis of the vessel, while platelets are prone to increase their presence near the vessel wall due to radial migration^8,9^. Erythrocytes help bring platelets to the surface of an injured vessel wall by random collisions between RBCs and platelets, which allows platelets to move across flow streamlines in a form of “enhanced diffusion”^10^. Collisions with flowing RBCs potentiate the molecular motion of platelets enhancing their ability to interact with the vessel wall^11^. Also, the size of erythrocytes may contribute to the platelet margination effect. In some mathematical models the main factor of thrombocyte migration towards periphery of the vascular wall is the size of platelets. When the space between two RBCs is smaller than the size of thrombocytes, they cannot reside in this volume and are “ejected” to the periphery of blood vessel^12^. Accordingly, high RDW may influence the motion and migration of RBCs as well as platelets since variation in shape and size of RBCs leads to a different dynamic behavior in the bulk flow. Therefore, the available volume for thrombocytes to be transported in the bulk flow, the size of the RBC-depleted region as well as the collision frequency between RBCs and thrombocytes in the near-wall region are affected and expected to lead to increased interaction with arterial wall.

We hypothesize that RDW is not only a marker of rapid progression of cardiovascular diseases, but it directly affects blood flow and interaction with the vascular wall. We assume that changes in blood flow caused by anisocytosis lead to interaction between the cellular components of blood and the vascular endothelium. This interaction causes overexpression of adhesive molecules in endothelial cells which may be an initiating factor for the development of inflammation in the vascular wall. Therefore in our study, we applied mathematical models and physical stimulation of blood flow in addition to traditional methods of molecular biology. We aimed to provide a link between increased interactions of blood cells with the vascular wall and red blood cell distribution width.

## Results

### RDW is associated with increased inflammation in atherosclerotic plaques

**Figure 1:**
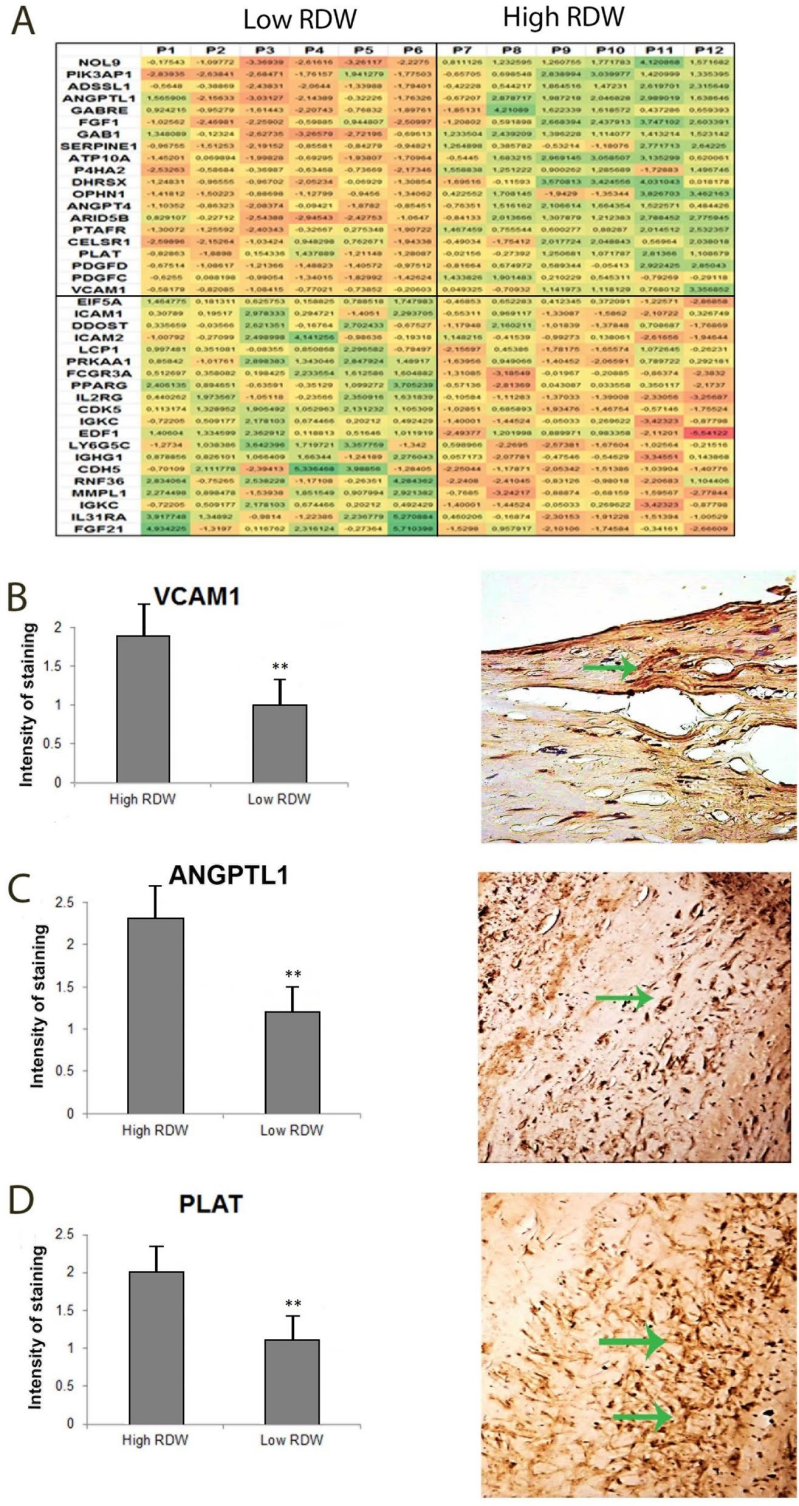
High RDW value is associated with increased expression of inflammation-related genes in human atherosclerotic plaques from carotid artery. (**A**) heat map from gene expression array. Microarray was performed on six patients with the low RDW (P1–P6) and six with high RDW level (P7–P12). First ten genes were positively correlated with RDW value, whereas next ten genes were negatively correlate with RDW; increased expression of (**B**) VCAM-1, (**C**) ANGPTL1, (**D**) PLAT, in samples form patients with normal and high RDW. Arrows indicate immunopositive cells. Graphs were created in Excel (Microsoft Office 2016, Microsoft, Albuquerque, NM, USA).

The study involved patients with distinct RDW values based on detailed hematological characteristics (Table 1). Microarray analysis of plaques from low- and high-RDW patients revealed statistically significant (p < 0.05) differences in expression of 2636 genes that were at least twofold up- or downregulated, of which, 587 genes were related to atherosclerosis and thrombosis. Using Panther database, a group of 39 representative genes related to atherosclerosis and involved in cytokine/chemokine-mediated signaling pathways, angiogenesis, cell adhesion, blood coagulation or inflammation was selected (Fig. 1A). There was a positive correlation between elevated expression of FGF1, GAB1, ANGPTL1, SERPINE1, P4HA2, PTAFR, CELSR1, PLAT, PDGFD, PDGFC and high RDW and inversely, these genes were downregulated in plaques from patients with low RDW. Furthermore, decreases in expression of VCAM1, EIF5A, ICAM1, ICAM2, PPARG, IL2RG, CDK5, EDF1 and IGHG1 were identified in samples from patients with low RDW, whereas high-RDW patients had increased expression of these genes. Differences in expression of VCAM-1, ANGPTL1, PLAT were confirmed on protein level by immunohistochemistry (Fig. 1B–D). To summarize, microarray results demonstrate that RDW is positively associated with intensified inflammation process in vascular wall. According to our hypothesis, found correlation between RDW and specified genes expression levels may be a result of more or less turbulent blood flow and consequently more or less sporadic communication of RBCs with endothelium.

**Table 1:** Haematological parameters of patients after carotid thromboendoarterectomy.

	Population		Low RDW		High RDW	
	Mean	SD	Mean	SD	Mean	SD
Age (years)	72.72	10.37	68.83	9.41	70.00	15.53
Gender (M/F)	50/48		6/0		4/2	
WBC (10^9^/l)	7.36	1.88	7.32	1.32	7.66	1.36
RBC (10^12^/l)	4.41	0.53	4.42	0.33	3.98	0.49
HG (g/dl)	13.38	1.71	13.93	1.18	11.27	1.20
HTC (%)	40.17	4.60	40.53	3.26	35.45	3.61
MCV (fl)	90.79	6.26	91.68	3.44	89.52	6.93
MCH (pg)	30.35	1.60	31.52	1.35	28.45	2.24
MCHC (g/dl)	33.26	0.88	34.37	0.69	31.78	0.29
RDW-CV (%)	13.93	1.18	12.17	0.23	17.17	1.43
RDW-SD (fl)	45.55	4.25	39.87	1.76	54.52	6.28
PLT (10^12^/l)	224.99	86.96	235.33	98.80	321.33	252.22
PDW (fl)	13.45	1.85	13.62	1.94	14.00	1.70
P-LCR (%)	32.16	6.34	32.83	7.85	32.63	5.80
PCT (%)	0.24	0.09	0.26	0.13	0.34	0.23
MPV (fl)	10.93	0.82	10.90	1.00	11.05	0.74
NEUTR (10^9^/l)	4.48	1.61	4.48	1.27	5.09	0.75
NEU% (%)	60.37	9.55	60.67	8.68	67.12	6.64
LYMPH (10^9^/l)	1.94	0.64	1.94	0.57	1.48	0.51
LYMPH% (%)	27.24	8.24	27.02	8.01	19.22	4.91
MON (10^9^/l)	0.68	0.23	0.72	0.19	0.71	0.24
MON% (%)	9.20	2.20	9.78	1.80	9.08	1.84

### RDW is associated with increased interactions between blood cells and vascular wall

**Figure 2:**
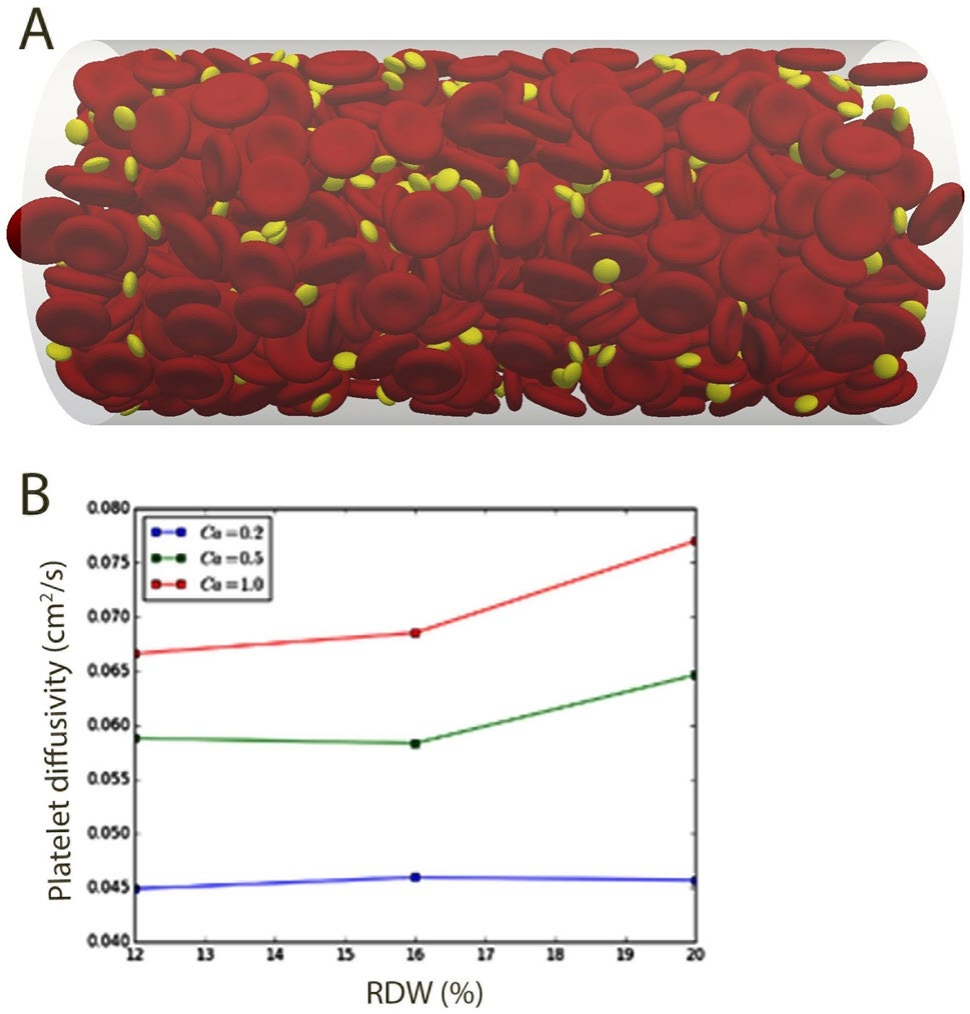
High RDW value correlates with increased interactions between blood cells and vascular wall. Flow simulation of RBCs (volume fraction 30%) and thrombocytes in a vessel having diameter and length of 42 μm and 126 μm, respectively (top). Platelet diffusivity for different RDW and distinct values of capillary number is shown in the bottom figure. Figure was created in Matlab (The MathWorks, Natick, MA, USA). The Capillary number (Ca = g a m/G, where g is the shear rate, a is the RBC radius, m is the dynamic viscosity of the liquid and G is the shear modulus of the cell membrane) is a non-dimensional number relating viscous to surface tension forces and used here to assess different stiffness properties of the red blood cells and its effect on distribution of RBC and platelets across the vessel diameter. (**A**) Represents settings for Ca = 0.2, corresponding to rigid RBCs. The figure would look different for different Ca as this would affect the interaction among the blood components, as shown by (**B**).

To elucidate the effect of anisocytosis on platelet migration towards vascular wall, lattice Boltzmann simulations of RBCs, blood plasma and platelets were performed. Three different RBCs volume distributions (Gaussian) having standard deviations of 12, 16 and 20% were investigated, maintaining a bulk RBCs volume fraction of 30% for all analyzed cases. The effect of RBCs deformability was considered by applying different shear modulus of 25, 50 and 126 μN/m, corresponding to capillary numbers (see Methods section for definition of 0.2, 0.5 and 1.0, respectively). An instantaneous picture of the distribution of RBCs and platelet distribution was presented as Fig. 2A, where the effective viscosity was found to be 3–4 times greater, as compared to plasma viscosity, and having a final average bulk velocity of 1.5–2 cm/s. Radial platelet diffusivity, obtained from the mean square displacement of platelets in the radial direction, expressed the level of mixing occurring in the cross-stream direction. A high radial diffusivity indicated a higher level of mixing. Furthermore, for rigid RBCs (Ca = 0.2), influence of RDW was less significant, whereas as the deformability of RBCs increased, elevated RDW led to increasing diffusivity (Fig. 2A,B). In conclusion, numerical simulations support our hypothesis that higher RDW is associated with increased interactions between cellular elements of blood as well as with vascular wall.

### High anisocytosis increases interaction between cellular elements of blood and vascular wall in a microfluidic system

**Figure 3:**
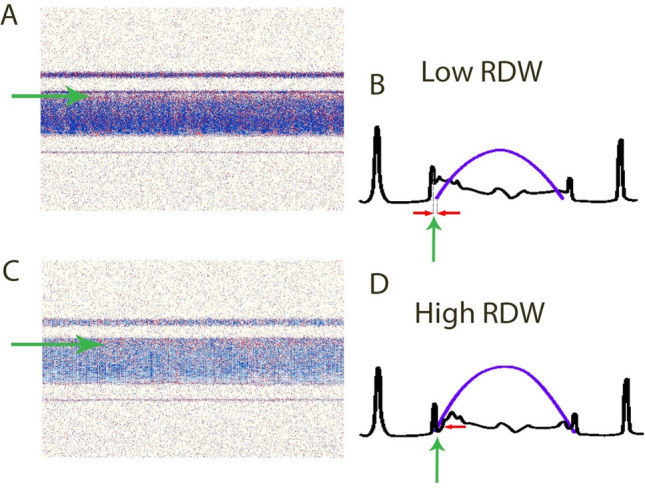
Optical coherence tomography of blood flow in a glass capillary comparing flow patterns of blood samples from patients with low and high RDW. The images show a distance between blood and glass wall among patients with low and high RDW. (**A,B**) Low RDW, (**C,D**) high RDW. Green arrows indicate space between glass wall and cellular components of blood. Left panels show Doppler OCT cross-sectional images of glass capillary with flowing blood. Right panels indicate drawings of cross-sectional profiles of the capillary for both: backscattered signal (black) and relative speed across the capillary lumen (blue). Imaging was performed with a Fourier-domain OCT setup. Data analysis was performed by using a joint spectral and time domain OCT method (STdOCT).

Human blood samples drawn from patients with low RDW (CV 13.34, SD 0.68) and high RDW (CV 17.14, SD 1.6) as shown in Table 2, were pumped through microfluidic channels. Analysis of blood cell distribution and velocity profile by doppler optical coherence tomography (OCT) indicated that space between blood cells and vascular wall was lower if RDW value was high (28–29 px), as compared to the results for normal RDW (30–32 px) (Fig. 3).

**Table 2:** Haematological characteristic of patients used for microfluidic analysis with optical coherence tomography.

Variable	RDW low		RDW high	
	Mean	SD	Mean	SD
Age (years)	45.40	19.76	67.71	12.27
Gender (M/F)	2/3		2/7	
WBC (10^9^/l)	5.42	0.70	8.67	3.87
RBC (10^12^/l)	4.52	0.58	4.90	0.40
HB (g/dl)	13.72	0.95	14.46	0.73
HT (%)	40.72	2.58	44.74	2.50
MCV (fl)	90.92	9.23	91.50	4.28
MCH (pg)	30.58	2.65	29.59	1.79
MCHC (g/dl)	33.68	0.55	32.34	1.18
RDW-CV (%)	13.34	0.68	17.41	1.60
RDW-SD (fl)	43.84	3.53	57.90	5.12
PLT (10^9^/l)	251.80	36.09	222.14	49.50
PDW (fl)	13.40	2.38	13.00	1.70
P-LCR (%)	32.84	8.70	31.97	8.53
PCT (%)	0.28	0.05	0.25	0.06
MPV (fl)	11.00	1.12	10.77	1.09
NEUTR (10^9^/l)	2.85	0.36	5.12	2.12
NEU% (%)	52.90	6.19	67.96	12.22
LYMPH (10^9^/l)	1.89	0.45	1.25	0.46
LYMPH% (%)	34.72	5.83	17.21	9.75
MON (10^9^/l)	0.51	0.09	0.79	0.15
MON% (%)	9.42	1.16	8.51	3.43
EOS (10^9^/l)	0.32	0.33	0.18	0.13
EOS% (%)	2.42	1.77	2.74	1.89
BASO (10^9^/l)	0.01	0.01	0.04	0.05
BASO% (%)	0.40	0.16	0.55	0.36

### RDW is associated with changes of blood flow pattern in mouse carotid arteries

**Figure 4:**
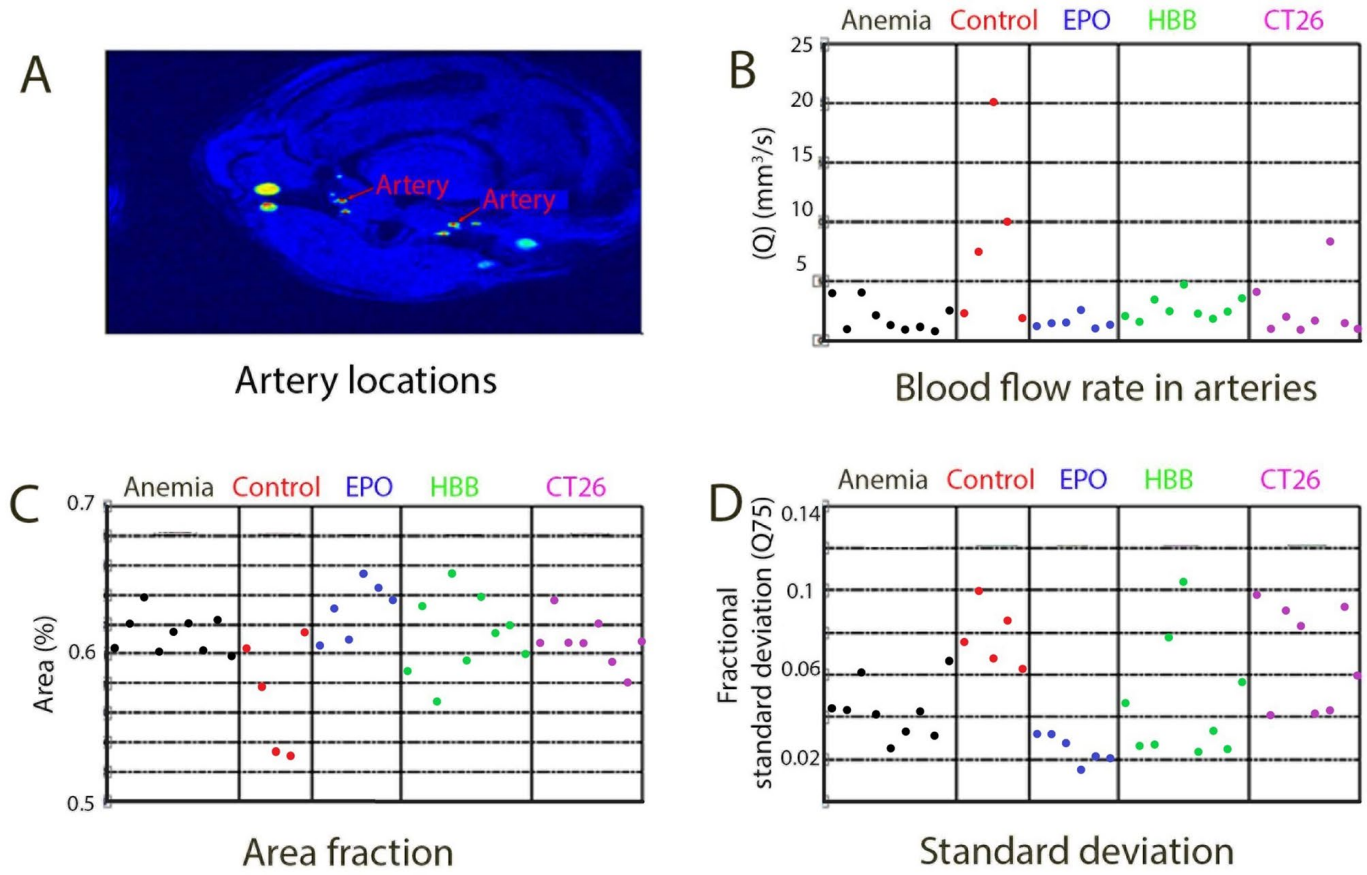
Measurements of blood flow in mouse carotid arteries with MRI indicate abnormal blood blow in mice with increased RDW. (**A**) Localization of vessels. (**B**) Maximum spatio-temporal volumetric flow rate through the external arteries. (**C**) Maximum spatio-temporal area fraction in which 75% of the total volumetric flow rate through the external arteries is found. (**D**) Temporal standard deviation over one cardiac cycle of the area fractions for 75% of the total volumetric flow through the external arteries. Graphs indicate measurements for anemic mice (Anemia), control mice (Control), Epo-treated mice (EPO), transgenic mice thalassemic Hbb (HBB) and mice with implanted CT26 colon cancer (CT26). Each dot represents one measurement of a different animal. Figure was created in Matlab (The MathWorks, Natick, MA, USA).

To investigate the relationship between RDW and the shape of the blood flow profile in carotid arteries, blood flow was measured by 9 Tesla MRI in distinct animal models of high RDW: mice with induced anemia (n = 9), mice with implanted murine colon cancer CT26 cells (n = 8), mice treated with erythropoietin (n = 6) and transgenic mice thalassemic Hbb homozygotes. (n = 9). As a control, BALB/c mice (n = 5) were used. Data on blood morphology of evaluated animals were included in Table 3. Digital image processing was used to extract information on blood flow in the arteries and veins of the cerebellum (Fig. 4A). Total flow rate, shape of the velocity profile and the variation of the velocity profile over the cardiac cycle, for all individual mice respectively were presented as Fig. 4B–D. Flow rate was lower in almost all mice with increased RDW as compared to the control (Fig. 4B). Moreover, area fraction of the cross section of the vessel containing 75% of the flow rate was lower and close to the value for a parabolic profile (0.51) of the control. In mice with increased RDW, the area fraction was higher, in turn an indication of a more sluggish velocity profile. Standard deviation of the area fraction over the cardiac cycle indicated that the variation of the velocity profile was less considerable for the anemic and EPO-treated mice which means the shape of the velocity profile displayed less variation during one cycle (Fig. 4D). However for HBB and CT26 group we observed more variation during one cycle. In general, MRI results describing structures of the blood flow in carotid arteries indicated apparent relationship between anisocytosis and intermittent or turbulent blood flow.

**Table 3:** Hematological parameters of mice that were used for analysis of blood flow in carotid arteries.

Parameter	UNIT	Control		EPO		Tumor CT26		HBB		Anemic	
		Mean	SD	Mean	SD	Mean	SD	Mean	SD	Mean	SD
WBC	10^9^/l	4.96	1.70	3.85	1.41	2.93	1.02	4.63	0.90	2.72	0.87
RBC	10^12^/l	6.00	1.69	6.35	1.31	5.27	1.33	8.33	0.90	5.76	1.58
HGB	g/l	6.23	1.57	99.33	20.37	5.48	1.11	7.80	0.53	6.73	1.55
HCT	l/l	0.30	0.08	0.28	0.06	0.28	0.07	0.41	0.04	0.35	0.08
PLT	10^9^/l	333.86	264.76	465.17	195.00	194.13	145.48	450.13	206.29	212.44	212.29
MCV	fl	50.14	0.69	43.90	1.87	53.25	1.67	48.75	2.92	51.00	1.00
MCH	pg	0.93	0.32	15.65	0.50	1.05	0.08	0.94	0.04	1.00	0.01
MCHC	g/l	18.03	7.44	356.67	6.74	19.80	1.83	19.00	0.61	19.56	0.34
RDW	L %	11.49	0.48	17.60	1.70	12.13	0.53	12.73	0.25	12.88	0.37
MPV	fl	6.44	0.47	7.00	0.18	6.15	0.57	4.83	0.13	5.27	0.51

### High RDW correlates with development of atherosclerotic lesion in the vessels

**Figure 5:**
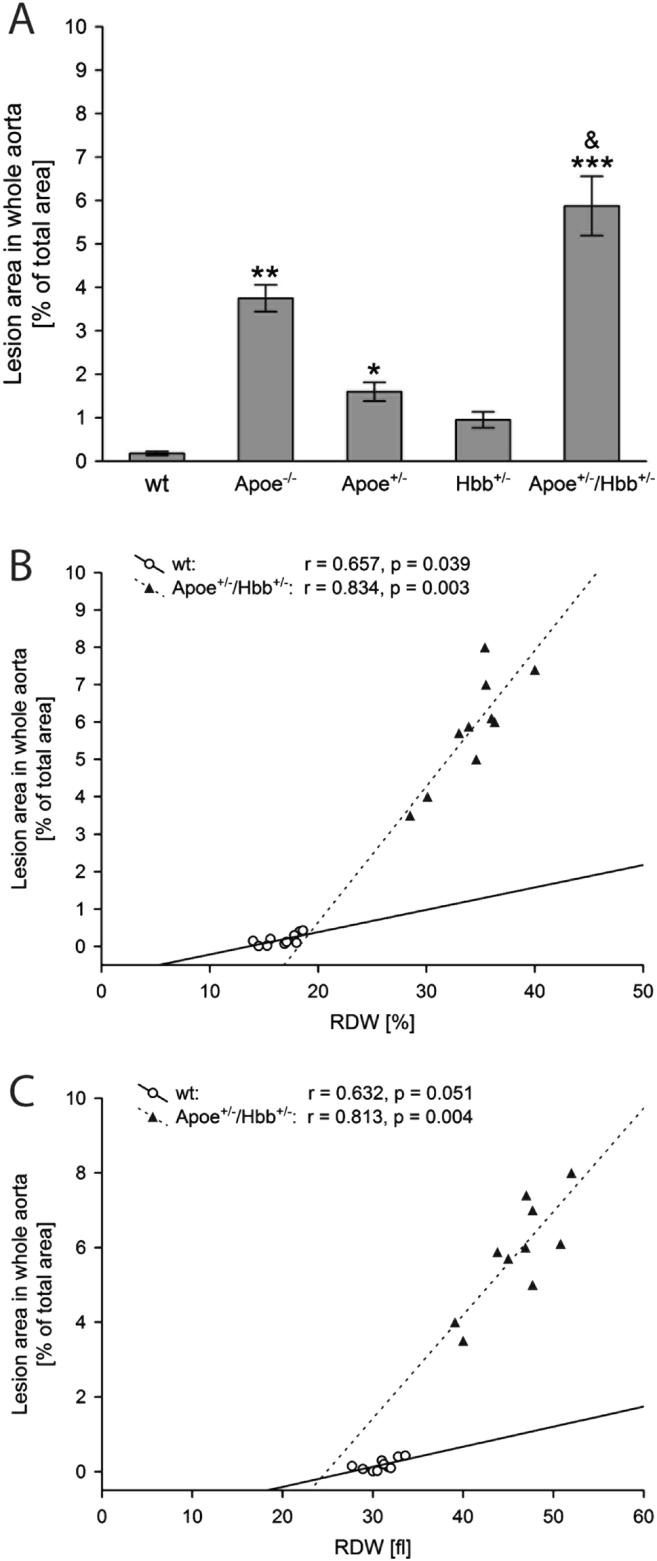
Increase in variation of red blood cells correlates with increased size of vascular lesion in mouse model of atherosclerosis. The mean (± SEM) lesion areas per each genotype. Post hoc comparisons between transgenic genotypes and control C57BL6/J mice in each time point are indicated by * (**A**). Regression analysis shows the correlation of lesion area in whole aorta versus %RDW (**B**) and RDW_fl_ (**C**) parameters. Each dot represents one mouse. Legend: wt: filled triangle; ApoE^+/−^/Hbb^+/−^: open circle. Graphs were created in Statistica 10 (StatSoft, Tulsa, OK, USA).

To obtain animal model with congenitally increased anisocytosis, ApoE^−/−^ mice were crossed with thalasemic Hbb^+/−^ mice, both having high RDW values. Parental generation of Hbb^+/−^ and ApoE^−/−^ mice as well as wild type C57BL/6J strain were used as a control. To strenthen the genotype effect related to abnormal RDW and increased propensity to develop atherosclerosis, all animals from each experimental group (control, parental and F1 generations) were additionally on a high cholesterol diet for 10 weeks. Next, aortas were isolated, stained and analysed to determine size of atherosclerotic lesions (Fig. 5A). Knockout of ApoE gene led to the most apparent atherosclerotic lesion manifestation as compared to the rest of control groups or the offspring following crossing. However, the effect observed in ApoE^−/−^ mice was further enhanced by high RDW and blood morphology parameters (Fig. 5B,C, Table 4). Therefore, high RDW induces inflammatory reaction associated with the development of arterial atherosclerotic plaque.

**Table 4:** Hematological values in transgenic mice and control group. One, two or three characters represent p ≤ 0.05, p ≤ 0.01, or p ≤ 0.001, respectively.

Parameter	Age [weeks]	Genotype				
		C57BL6J	Apoe^−/−^	Apoe^+/−^	Hbb^+/−^	Apoe^+/−^/Hbb^+/−^
RBC [10^6^/μl]	16	6.28 ± 0.72	7.35 ± 0.28	7.15 ± 0.42	3.06 ± 0.64**	7.48 ± 0.41^&&^
	24	6.69 ± 0.37	8.03 ± 0.51	7.76 ± 0.37	4.98 ± 0.96*^#^	6.56 ± 0.46
HGB [g/dl]	16	13.10 ± 1.47	15.41 ± 0.31	14.35 ± 0.89	5.51 ± 1.52***	12.92 ± 0.69^&&^
	24	12.11 ± 0.63	15.20 ± 1.11	14.70 ± 1.40	8.45 ± 1.14*^#^	10.75 ± 0.52
HCT [%]	16	30.95 ± 2.61	40.54 ± 0.51*	35.22 ± 1.98	12.94 ± 3.21**	26.17 ± 2.31^&^
	24	29.96 ± 1.39	38.04 ± 1.98	33.99 ± 3.45	18.32 ± 3.35*	22.27 ± 0.87
MCV [fl]	16	49.67 ± 1.45	50.99 ± 1.02	49.25 ± 0.63	36.02 ± 0.99**	34.99 ± 1.03**
	24	48.03 ± 0.79	47.51 ± 2.48	43.20 ± 2.81	36.75 ± 3.09*	34.25 ± 1.11**
MCH [pg]	16	20.87 ± 0.61	19.25 ± 0.35	20.07 ± 0.49	17.84 ± 1.25	17.50 ± 0.28*
	24	19.37 ± 0.23	18.51 ± 0.48	18.74 ± 1.08	16.95 ± 0.92	16.43 ± 0.39*
MCHC [g/dl]	16	42.08 ± 1.41	39.30 ± 0.51	40.75 ± 1.44	49.15 ± 3.34	49.34 ± 1.65*
	24	40.27 ± 0.33	39.23 ± 0.47	43.44 ± 0.79	46.37 ± 1.69	48.13 ± 0.64*
RDW [fl]	16	31.50 ± 0.68	36.15 ± 2.14	33.17 ± 0.98	47.31 ± 0.42***	48.85 ± 1.94***
	24	30.64 ± 0.39	35.64 ± 2.66	34.06 ± 2.10	41.05 ± 3.85*^#^	44.57 ± 2.04**
RDW [%]	16	16.29 ± 1.02	19.25 ± 0.23	17.22 ± 0.73	33.83 ± 0.84***	35.86 ± 0.43***
	24	16.56 ± 0.44	18.52 ± 0.48	20.64 ± 2.32	30.07 ± 2.75**^#^	33.52 ± 1.18***

*In comparison to C57BL6J genotype.

^&^In comparison to mice with Hbb^+/−^ genotype.

^#^Mice in 16-week vs. mice in 24-week.

## Discussion

Results from extensive translational research using fluid physics, optics, and biological models indicate that variation in size of blood cells affects the physics of blood flow including changes in flow characteristic such as turbulence and level of interactions between cellular elements of blood and vascular endothelium. These events in turn may lead to increased inflammation in the vessels, atherosclerosis and thrombosis.

Atherosclerotic lesions develop as a consequence of the mode of blood flow which is influenced—among others factors, by viscosity and density, shaped by the migration and transport of cells and macromolecules. Using in silico lattice Boltzmann method (LBM), we have demonstrated that rise of RDW value alters radial migration of the platelets and may thus facilitate their interaction with the vessel wall. Lattice Boltzmann method has become one of the most popular methods in computational fluid dynamics, it simulates blood flow in a complex microvascular network and allows to identify determinants of interactions between circulating blood elements and endothelium. It was shown previously—using LBM, that changes in vessel geometry like curvatures and bifurcations result in increased fluid viscosity and elevated force from blood elements to vascular wall, inducing changes in endothelial cells that promote atherosclerosis^13^. This mechanism explains typical localizations of atherosclerotic plaques in human arterial tree.

Using the same in silico approach, we have demonstrated that elevated RDW value potentiates the interactions of blood components, as RBCs and thrombocytes, with the vascular wall. In recent years, there has been accumulating evidence that platelets play roles beyond thrombosis and hemostasis, e.g., in inflammatory processes, infection and cancer^14^. Therefore, it is plausible that association between RDW and different clinical outcomes may also involve platelet-related mechanisms.

We studied flow patterns of blood samples from subjects with different RDW values in microfluidic channel system. To study this process we have employed Doppler optical coherence tomography (DOCT), which enables simultaneous visualization of the structure and blood flow. The method can be used in animal models of different human diseases to assess flow patterns and interactions of blood morphotic elements and vascular wall^15^. Presented data show that high RDW value led to shrinkage of space between flowing blood cells and vascular wall, in consequence promoting direct contacts between morphotic elements of blood and capillary. Samples of blood with low and normal RDW value presented similar, but greater than high RDW samples, distance between blood cells and vascular wall. These in vitro findings are in concordance with in silico analysis performed using lattice Boltzmann method. Therefore, results from our studies suggest that elevated RDW value may accelerate interaction of platelets with endothelium contributing to the development of atherosclerosis as well its clinical complications.

Despite the fact, that we only show the association of high RDW with increased interactions of blood cells with the vascular wall we think that we have found a missing piece to theory about the causativity of the RDW. It is known that vascular cell mechano-transduction of flow dynamics and cell to cell interactions may trigger cytokine release and cross-talk between cell types within the artery^16^. It has been demonstrated, that leukocytes are transported toward the vessel wall likely via hydrodynamic interactions with the relatively smaller and more flexible erythrocytes. In order to initiate rolling, circulating leukocytes must marginate to contract the vessel wall. Leukocyte margination has been attributed to the ability of RBC aggregates to exclude the WBCs from the bulk solution^17^ and also to the interactions between individual white and red blood cells at bifurcations or postcapillary expansions^14,18^. Random and lateral displacements of RBCs (high RDW) may produce repeated collisions with lymphocytes located near the wall and consequently promote increased incidence of direct interactions of lymphocytes with vascular-wall and initiation of the inflammatory process. Adhesion modeling demonstrated, that a lymphocyte, when adhere to the vascular wall is much easier pulled off the wall by a single red blood cell than “a train of erythrocytes” due to downward forces of the RBC series passing the attached lymphocyte and pushing it down into the wall^19,20^. The higher variation in size of the RBCs together with lower vessel diameter seems to facilitate forming of “a rope” rather than equally dispersed single cells.

Described observation of RBC’s role as a helper or reinforce stabilizer in initial arrest or solid attachment to the endothelium mediated by VCAM-1 and ICAM-1, respectively, is consistent with results obtained in another study^20^. The authors prepared platelet-free blood containing T-lymphocytes at 32% hematocrit and tested the flow medium effect on lymphocyte capture of the endothelial monolayer in vitro. Interestingly, the efficiency of cell capture approximately doubled at moderate shear stress with the addition of erythrocytes. The addition of RBCs also increased the fraction of rolling cells as compared to adherent cells, and rolling velocities were comparable to those measured in vivo^20^. Melder et al. postulated that a mechanism of the enhancement effect of RBC could rely on the outward dispersive effect of RBCs produced by cell interactions at physiological hematocrits during flow in a narrow tube. Then, random and lateral displacements of RBCs may produce repeated collisions with lymphocytes located near the wall and consequently promote increased lymphocyte-wall collisions. Moreover, RBCs aid in rapid lymphocyte recapture after detachment from the endothelium, a phenomenon of even greater significance under conditions of high arterial shear stress^20^.

Presented data show increased interaction of blood morphotic elements with the vascular wall in conditions of high RDW. We believe that this interactions are also a source of oxidative stress in the vessel and initiate endothelial damage. RBC collision with the aortic wall has recently been reported as an important source of the oxidation process in early human aortic atheroma^21^. Non-laminar flow causes loss of this property and brings the RBCs close to the arterial wall. The same is observed in conditions of high RDW. With time, anucleate erythrocytes progressively expose phosphatidylserine on their external membrane, as a signal of senescence recognition. Senescent RBCs are frail and more sensitive to hemolysis and their detrimental cytotoxicity on the arterial wall is mainly mediated by hemolysis and the powerful oxidative capacity of heme-ferrous iron which may initiate endothelial cell damage. For instance, the focal external application of FeCl_3_ is a usual model to induce experimental clotting in arteries. This model involves the inward cellular transport of FeCl_3_ towards the endothelium and is dependent on local hemolysis of circulating RBCs^22,23^. Formation of endothelial RBC aggregates, hemolysis, and loss of endothelium precede platelet activation and thrombus formation in this model^23,24^. Sites of the bloodstream reattachment are likely the areas of frequent RBC collisions that add more to the pre-existing physical forces also due to RBC overrepresentation in the blood as compared with other types of blood cells and change the gene expression pattern of the endothelial cells. Therefore, the specific sites of plaques formation is an effect of synergistically acting RBCs constituting dominant cell type component in the circulation system, other morphotic elements of the blood, and hemodynamic forces.

A portion of key information of the increased RDW role on blood rheology and atherosclerosis development, as well as another example of RBCs contact with the vascular wall, provides studies on sickle cell disease^25,26^. In opposition to normal RBCs, sickle RBCs demonstrate polymerization of sickle hemoglobin towards the cell surface creating several sites of abnormal cytoadherence to human endothelial cells in vitro and hypoxia enhanced this adhesion^26^. Although in the in vivo systems, sickle RBCs attachment predominates in postcapillary venules with the low shear stress near the wall of ~ 0.1 Pa, the performed simulations by Papageorgiou indicate that RBC shape heterogeneity (and RBC deformability) facilitate contact with the vascular wall^26^. One of the earliest in vitro studies on the sickle cell disease also demonstrated, that sickle erythrocytes are able to induce expression of vasoconstrictor endothelin-1 (ET-1) in primary cultures of human umbilical vein endothelial cells (HUVEC), therefore regulating local vasomotor tone directly^25^. ET-1 has been described to play a role in fibrosis, angiogenesis, and inflammation. Moreover, ET-1 stimulates endothelial angiotensin-converting enzyme (ACE) activity and secretion of aldosterone participating in hypertension development^27^.

We did not study the mechanism contributing to variability of RBC size. In humans, RDW variations are related to different types of anemias but there is also an association with age, gender, genetic factors, renal and liver function, blood pressure, metabolic factors and inflammation^1^. Many of these conditions are potent cardiovascular risk factors, therefore relation between RDW and atherosclerosis may be incidental not causal. However, the hypothesis that high RDW may contribute to the development of vascular lesions cannot be rejected and we have provided supporting evidence in our study.

First, in ex vivo setting without any additional interfering factors like inflammation, high RDW promotes potentially atherogeneic interactions between the blood elements themselves and vascular wall. This has been shown in in silico experiments (lattice Boltzmann method) as well as in microfluidic channels measuring flow patterns of blood samples differing in RDW values.

Second, we applied different mouse models with high RDW induced by anemia, transplanted colon cancer cells, erythropoietin treatment or thalassemia. Some of these experimental setting may also include processes like inflammation, prothrombotic conditions that may contribute to vascular lesions and affect pattern of blood flow. However, measurement of blood flow in carotid arteries using non-invasive magnetic resonance imaging showed intermittent or turbulent blood flow. This pattern of blood flow is unequivocally associated with blood-vessel wall interactions inducing atheroma formation. We have also studied crossbreds of thalassemic high RDW mice with atherosclerosis-prone ApoE^−/−^ strain. Anisocytosis accelerated development of lipid plaques in aortas of ApoE deficient mice as compared to animals without thalassemic gene.

Third, we analyzed samples of atherosclerotic plaques removed during carotid thromboendoatherectomy in patients with significant carotid stenosis. Numerous data demonstrate that when local properties of viscosity and density of blood are altered, along with the flow pattern, local shear stress can be modified. Shear stress affects atheroprotective mechanisms^18,19^, for example, these local hemodynamic forces induce production of antioxidant enzyme, superoxide dismutase, or upregulates the expression of nitric oxide synthase. As a consequence of nitric oxide (NO) overproduction, VCAM-1 expression decreases by inhibition of nuclear factor kappa beta (NFkB) that prevents platelet clumping and inflammation^20^. Our analysis of human atherosclerotic samples showed that in patients with anisocytosis, there is an increased expression of VCAM-1 that facilitates plaques formation and mediates adhesion of blood cells, including monocytes, to vascular endothelium. Once adhered, the monocytes penetrate vascular endothelium and infiltrate the intima in the process of diapedesis that requires a chemoattractant gradient such as monocyte chemoattractant protein 1 (MCP-1)^28^. This is one of important steps in the development of atherosclerotic plaque. Another factor is a shear stress. Shear stress-dependent elevation of platelet-derived growth factor A (PDGF-A) and B (PDGF-B) mRNA in cultured human umbilical vein endothelial cells (HUVEC) has been described, as well as increased endothelial secretion of tissue plasminogen activator (PLAT) induced by blood flow-mediated shear stress^29^. Similarly in our microarray study we have observed elevated expression of those genes in patients with high RDW level. It has been also documented that oscillatory shear stress limits fibroblast growth factor receptor 1 (FGFR1) expression in endothelium (protective signaling pathway preventing atherosclerosis) and stimulates transforming growth factor β (TGF-β) signaling. Both pathways have been proved to play crucial role in endothelial-mesenchymal transition of endothelium and atherosclerosis progression^30^. Hence these findings propose that the flow characteristics such as level of velocity fluctuations appearing in the flow field might induce a variety of molecular responses in ECs that result in sparing of the straight parts of the arteries and formation of lesions at the curvatures. Although mentioned genes are known to have impact on atherosclerosis development, for the first time we have found in human plaques a correlation between RDW and altered expression of genes important in pathogenesis of atherosclerosis.

## Conclusion

We have applied different experimental approach (in silico, ex vivo, animal models and human study) to study the effect of high RDW on blood flow pattern and interactions between blood elements and vascular wall. Our results show that RDW accelerates interaction between blood elements (platelets and blood cells) which is associated with development of atherosclerosis. These data support the hypothesis that RDW may increase cardiovascular risk directly affecting mechanisms related to atherosclerosis.

### Limitations of the study

Lack of arterial samples of the patients with different RDW values without any concomitant medical conditions, therefore the effect of other factors cannot be definitely excluded.

Lack of in vitro study with cultured endothelial cells exposed to shear stress and different components of blood of high vs. low RDW to determine the early changes in gene expression upon controlled, forced physical collisions of RBCs of different sizes.

Mouse models used also include concomitant factors (inflammation, increased procoagulant activity) that can affect studied parameters like pattern of blood flow.

## Methods

### Microarray analysis of human samples

To the study 98 patients were recruited. From the population of 98 patients, those with lowest and highest RDW value were included in further analysis. Microarray analysis was performed on 12 carotid plaques derived from 6 patients with low and from 6 patients with abnormal parameter value > 16. Gene expression profiles were analyzed using an Affymetrix (Santa Clara, CA, USA) at Karolinska Institute (KI). We found statistically significant (p < 0.05) differences in 2636 genes expressions that were up or downregulated (at least twofold change) in plaques derived from patients with high RDW as compared to genes expression pattern found in samples from normal RDW patients. Next, those genes were filtered using Panther DB to choose those, which are known to be involved in pathways associated with pathogenesis of atherosclerosis. Moreover, proteins encoded by those genes were localized in aorta using immunohistochemistry technique. Necessary ethical approval (No 2011411-311) was obtained prior to material collection from patients that were planned to have carotid endarterectomy as standard treatment for their atherosclerotic lesions.

### Mouse model of blood flow in carotid arteries

To analyze differences in blood flow through cerebral arteries in BALB/c mice with high RDW induced under distinct conditions, several manners of anisocytosis induction were undertaken. To trigger inflammation-mediated anisocytosis, mice (n = 10) were injected with 3 × 10^6^ of murine colon cancer CT26 cells that resulted in high RDW within 2 weeks. Second group of mice (n = 10) received erythropoietin for 4 subsequent days intravenously and the third group of animals (n = 10) was on a low iron diet for 4 weeks and had 0.6 ml of blood drawn regularly (once a week, for a period of 3 weeks) to trigger anemia and induce inflammation-independent anisocytosis. Control mice (n = 10) constituted healthy, untreated BALB/c mice. All animals were on normal diet except for one group fed iron-deficient chow. Next, all mice, exposed to different factors contributing to high RDW value, had anisocytosis confirmed by blood tests and MRI performed using a 9 Tesla at the Karolinska Institute to observe blood flow in the arteries and veins of the cerebellum and to compare changes in blood flow between groups. Animal studies were conducted according to the guidelines of the Declaration of Helsinki, and approved by the Institutional Review Board of the Karolinska Institute (protocol code N12/13, date of approval—11 April 2013).

### Simulation of blood flow in microchannels

Simulation of blood flow was performed using the lattice Boltzmann method (LBM), in-house solver available at KTH Mechanics. The aim of using lattice Boltzmann (LBM) was to assess how variance in RDW influences the transport of platelets within the vessel. LBM is one approach to numerically assess the transport and distribution of particles and is commonly used in fluid dynamics to study dense particle suspension flows (such as blood flow). LBM is in this study used to assess the interaction among the blood components as well as the distribution of the different components within the blood vessel. The numerical setting provides an opportunity to study the blood components individually, not possible experimentally for a flow situation as considered here. Analyzed vessel had diameter and length of 42 μm and 126 μm, respectively. The volume fraction of RBCs investigated was 30%. To capture the effect associated with RDW, a deviation in RBCs volume corresponding to 12, 16 and 20% with respect to mean RBC volume (80 μm^3^) was applied (maintaining a 30% bulk RBC volume fraction). Moreover, the shear modulus of the RBC was changed to consider possible variability in RBC stiffness. The platelets were considered rigid and spherical objects with a diameter of 2 μm. To initiate the simulations, a random distribution of RBCs and platelets was used, from which the motion of the cells was tracked over time. A constant volume force was applied in the flow direction corresponding to the pressure drop in a flow of solely blood plasma having a velocity of 6 cm/s. At the boundaries, a periodic inlet–outlet condition was applied along with no-slip at the vessel wall and cell surfaces.

The radial platelet diffusivity was obtained from the mean square displacement of platelets in the radial direction, defined as:

$$D_{rr} = \frac{{\left\langle {r(t) - r(t_{0} )} \right\rangle }}{{t - t_{0} }},$$

where r_t_ is the radial position at time t, and $\left\langle \cdot \right\rangle$ denotes the average over all platelets in the simulations. Moreover, the capillary number was used to quantify the shear modulus of the RBCs, defined by the formula:

$$Ca = \frac{{\gamma_{w} \mu_{p} a_{RBC} }}{G},$$

where γ_w_ is the wall shear rate, μ_p_ is the plasma viscosity, a_RBC_ is the mean RBC radius and G is the shear modulus. Increasing the capillary number, increases the deformability of the RBCs.

### Blood flow in microchannels

We measured velocity profiles of blood flow in microchannels with Doppler optical coherence tomography (DOCT). Venous blood was drawn from patients and samples with high and low RDW values were studied using DOCT and microfluidic channels as previously described^31^. The OCT signal was detected by a custom-designed spectrometer containing a collimating lens (Schneider Kreuznach Tele-Xenar, 2.2/70 mm, Germany), a volume holographic diffraction grating (1200 LP/mm, Wasatch Photonics, USA), a telecentric f-theta lens (effective focal length 79.6 mm, Sill Optics), and a 12-bit CMOS line-scan camera (spl4096-140 km, Basler Sprint, Germany). Analysis of intensity of dynamic backscattering of moving particles enables to asses velocity and structure of blood flow simultaneously^32^.

### Magnetic resonance imaging (MRI)

MRI studies were conducted on mice from different groups to determine blood flow velocity and shear stress in the carotid veins, especially at the vessel bifurcations. Mice were anesthetized and prepared for scanning according to the adopted protocol. MRI was performed in collaboration with Peter Damberg at the Karolinska Experimental Research and Image Center (KERIC) facility using a Varian 9.4 T scanner with a 30 cm bore.

### Immunohistochemical analysis of atherosclerotic tissues

Immunohistochemical analysis was performed using previously established method^33^. Following primary antibodies were used: anti-VCAM-1, anti-ANGPTL1 and anti-PLAT (Abcam, UK). As secondary antibody anti-rabbit/mouse ImPRESS universal staining kit (Vector Laboratories, CA, USA) was applied. Sites of reactions were visualized as previously described^34^. Intensity was calculated according to 3 points scoring system (0, no staining; 1, weak; 2, mild; 3, strong staining).

### Animals and genotyping for analysis of atherosclerosis

Transgenic mouse lines: B6.129P2-Apoe^tm1Unc^/J (ApoE^−/−^) lacking apolipoprotein E and B6.129P2-Hbb-b1^tm1Unc^ Hbb-b2^tm1Unc^/J (Hbb^+/−^) with knockout of genes encoding both hemoglobin chains were purchased from Jackson Laboratory (USA). For the atherosclerosis experiments, ApoE^−/−^ mice were mated with the Hbb^+/−^ mice. Male double transgenic mice (Hbb^+/−^ × ApoE^+/−^) were compared to age-matched male ApoE^−/−^, Hbb^+/−^, and control C57BL6/J mice. Genotyping was performed by PCR, using tail-tip DNA according to the protocol provided by the Jackson Laboratory. Animals were fed a standard chow for 8 weeks. At 9 weeks of age, mice from each experimental group started a high fat diet (with 0.5% cholesterol supplementation, Altromin, Germany) and continued 24 weeks to induce hyperlipidemia and atherosclerosis.

### Collection of material

At 32 weeks, mice were anesthetized and blood samples were drawn from the heart into EDTA-coated tubes to prevent coagulation. Then, through the left ventricle, the animal was perfused transcardially with phosphate-buffered saline until the eluent from the right auricle became clear, followed by 10% buffered formalin infusion. Finally, aortas were placed in the fixative at 4 °C overnight.

### Blood sample preparation and analyses

Blood was collected directly from heart of anesthetized mice to EDTA coated syringes. Each blood sample was divided in two equal parts. First part was used for complete blood count by hematology analyzer (Diatron^®^, Vienna, Austria). Second part of blood was centrifuged at 1000×*g* in 4 °C for 10 min, then plasma was collected and stored at − 80 °C until further analysis.

### Oil Red O staining for identifying lipid deposits

The aorta (from aortic valve to iliac bifurcation, including three branching arteries at aortic arch) was dissected away intact from the dorsal wall. Aortas were fixed in 4% formaldehyde, opened longitudinally, pinned onto black wax plates and stained. The aorta was then rinsed with 70% ethanol for 5 min, then incubated for 8 min in filtered Oil Red O stain solution (a fat-soluble dye that stains triglycerides and protein-bound lipids red) containing 0.5% Oil Red O (Sigma-Aldrich, Germany), in isopropanol, then the specimen was rinsed in distilled water to clear redundant staining. Next, stained aorta was placed in PBS. The total Oil Red O-stained lesion area was quantified using ImageJ software, version 1.38× (National Institutes of Health, Bethesda, MD). The final data are expressed as a percentage of positive-staining areas relative to the total aortic area.

### Statistical analysis

Statistical analysis was performed using Statistica 7.0. Data obtained from animal experiments were analyzed using Student’s t-test. For comparing the data gathered during human studies, we performed ANOVA, followed by least significant differences (LSD) post-hoc test. We considered the results as statistically significant when p-value was lower than 0.05. All data presented on graphs are mean ± standard deviation (SD).

### Ethical statements

Animal studies were conducted according to relevant guidelines and regulations. Experimental protocols were approved by the Institutional Review Board of the Karolinska Institute (protocol code N12/13, date of approval—11 April 2013). All the information related to animal studies follows the recommendations in the ARRIVE guidelines.

Human studies were performed in accordance with the Declaration of Helsinki. Ethical approval No 2011411-311 was obtained from Commission for the Supervision of Research on People and Animals at the Central Clinical Hospital of the Ministry of Interior and Administration in Warsaw. Informed consent was obtained from all participants prior to material collection.

## Data Availability

The datasets used and/or analysed during the current study available from the corresponding author on reasonable request.
